# On the Various Operations Performed for the Cure of Stammering

**Published:** 1841-06-19

**Authors:** J. Trudeau

**Affiliations:** New York


*On the various operations performed for the cure of Stammering.* By J. Trudeau, M. D., of New York.

The great excitement caused in Europe by the operation for strabismus, has, in some degree, subsided, on account of the recent discovery of the curability of stammering, made by the celebrated Dieffenbach. In Paris especially, the new operation found many admirers, and, even as they termed it there, inventors.

MM. Baudens, Velpeau and Amussat have contested with the German surgeon the priority of invention. In fact, it was with it as with strabismus, every one begging a share of the discovery, or, at least, of improvement; and this being always the case with all new discoveries, we must not wonder at the severe attacks made upon the surgeon of Berlin. It seems, however, from the most impartial accounts, that a short notice was published in one of the Paris newspapers, mentioning that a German surgeon had succeeded in curing the affection called stammering, by means of *an incision of the tongue.* No further particulars were given. Upon that ground, Messrs. Amussat, Velpeau, and Baudens, together with Philips, a quack, styling himself one of Dieffenbach's private pupils, went to work, and a *few days* afterwards presented to the scientific institutions several patients, *perfectly cured.* In consequence, they all claim the title of discoverer.

So many claims were lately, on that subject, presented to the Academy of Medicine, and so many harsh words passed between the members, that it was decided that no further communications on the subject of *glossotomy* (the new consecrated term) should be received. The letter of Dieffenbach was in consequence read at the Academy of Sciences. From that, it appears that Dieffenbach attacks the tongue itself, while the French surgeons act upon the muscles of that organ.

Mr. D. has experimented in three methods:

1st. The horizontal transverse section of the *base* of the tonge.

2d. The subcutaneous transverse section of the *base* of the tongue.

3d. The horizontal section of the *base* of the tongue, with excision of a triangular portion.

To the last method Prof. D. gives a decided preference, as being *free from danger*, and more efficient. His first operation was performed on the 21st of January, 1841. In all the cases operated on (namely, fifty-nine) he never failed to effect a cure.

M. Amussat performed his operation on the 17th of February. His method is entirely different from M. Dieffenbach's. The first step of the operation is to make a complete division of the frænum; he then causes the patient to speak, and, if the speech is not free from hesitation, he divides the main body of the genio glossial muscles, above the sub-lingual glands. According to him, twenty-two stammerers were *glossotomized*, and radically cured.

Mr. Philips, of Liege, upon whom we place no confidence on account of his quackery, has had (according to himself,) astonishing success, but no one yet has seen his patients, nor has he published his operative mode.

M. Baudens divides the tendons of the genio glossial muscles at their insertion at the genial process with sharp scissors, plunged on each side of the frænum, close to the inferior maxillary bone.

According to our own views, M. D.'s method is far from being *entirely free from danger*, as we could relate a fact reported in the Gazette des Hôpitaux, in which he lost one of his patients, a medical student, of hæmorrhage; another one of a violent inflammation of the tongue extending to the fauces.

Some others experienced such serious accidents, that their lives were despaired of. The very idea of that operation seems to have frightened the French surgeons. Mr. Velpeau, one of the boldest of them, has attempted to effect by ligature the separation of the wedge-shaped portion, removed with the knife by Dieffenbach. Hæmorrhage and consecutive inflammation of the tongue is so much to be dreaded, that no prudent surgeon will give preference to that method.

We would give a decided preference to Baudens' method, over that of Amussat, or Dieffenbach, as being comparatively safe and of easy performance; but according to Mr. B. himself, it does not seem so efficient as Dieffenbach's. Its results, however, are sufficiently fair to encourage further experimentation.*

A summary of the various operations proposed for the cure of this affection, with their results and their appreciation, by the London and Westminster Medical Societies, will be found in No. 22 of the Examiner.—Eds. Exam.

* An English Physician has removed the tonsils and uvula in the same view, (cure of stammering,) and proposes that operation as an effectual substitute to glossotomy. To some cases of induration of these organs, improvement may possibly be obtained in the speech. In other cases I do not understand the propriety, nor the rational object of that operation, which, I must confess, seems to me perfectly absurd. J. T.

